# Chinese visceral adiposity index outperforms other obesity indexes in association with increased overall cancer incidence: findings from prospective MJ cohort study

**DOI:** 10.1038/s41416-025-03041-1

**Published:** 2025-05-10

**Authors:** Mengying Wang, Chi Pang Wen, Junlong Pan, Gege Sun, David Ta-Wei Chu, Huakang Tu, Wenyuan Li, Xifeng Wu

**Affiliations:** 1 Center of Clinical Big Data and Analytics of the Second Affiliated Hospital and School of Public Health, Zhejiang University School of Medicine, Hangzhou, 310058 Zhejiang China; 2 Institute of Population Health Sciences, National Health Research Institutes, Zhunan, Taiwan; 3 Graduate Institute of Biomedical Sciences, College of Medicine, China Medical University, Taichung, Taiwan; 4 MJ Health Management Center, Taipei, Taiwan; 5 Zhejiang Key Laboratory of Intelligent Preventive Medicine, Hangzhou, 310058 Zhejiang China; 6 National Institute for Data Science in Health and Medicine, Zhejiang University, Hangzhou, 310058 Zhejiang China; 7 School of Medicine and Health Science, George Washington University, Washington, DC USA

**Keywords:** Risk factors, Predictive markers

## Abstract

**Background:**

We assessed the associations of visceral adiposity indexes such as Chinese Visceral Adiposity Index (CVAI), Visceral Adiposity Index (VAI), Lipid Accumulation Product (LAP), waist circumference (WC), and waist-hip ratio (WHR) with overall and specific cancer incidence in a Chinese population.

**Methods:**

332,297 individuals from the Taiwan MJ cohort (1996–2007) were included. We utilized multivariable Cox proportional hazards models to examine associations of baseline visceral adiposity indexes and cancer incidences. Sex-specific CVAI, VAI, and LAP were calculated, incorporating WC and triglycerides levels. CVAI and VAI also included body mass index and high-density lipoprotein, with CVAI further incorporating age.

**Results:**

Higher CVAI was consistently associated with higher overall cancer incidence, with HRs of 1.45 (95% CI: 1.2–1.76) and 2.03 (95% CI: 1.52–2.72) for males and females, respectively, comparing the fifth quintile to the first. The HRs for WC were 1.27 (95% CI: 1.08–1.49) and 1.19 (95% CI: 1.01–1.40) for males and females, WHR was significantly associated with cancer risk in males (HR:1.28; 95% CI: 1.13–1.45), and LAP was significantly associated with cancer risk in females (HR: 1.25; 95% CI: 1.04–1.5). VAI was not associated with overall cancer incidence.

**Discussion:**

CVAI is a superior clinical biomarker for predicting cancer incidence in the Chinese population compared to traditional visceral obesity indices.

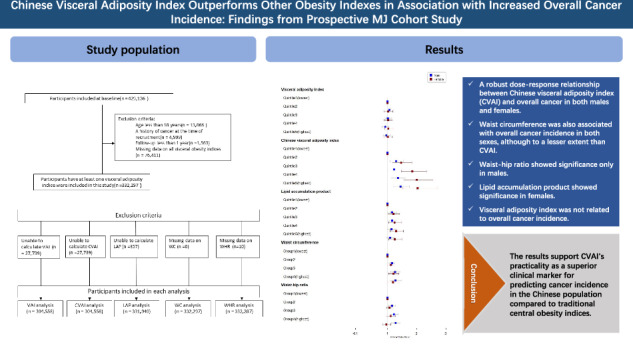

## Introduction

Obesity increases the risk of chronic diseases, including cancer [1]. According to the available data from the International Agency for Research on Cancer, obesity was a causal factor for 13 types of cancers, which include cancers affecting the colon, liver, kidney, and others [2]. In 2023, among 15.8 million Chinese adults, 34.8% were overweight and 14.1% were obese according to the Chinese BMI classification [3]. Obesity, especially abnormal visceral adipose tissue (VAT), could lead to a hormonal, metabolic, and inflammatory environment that promotes tumor development [4–6]. An expanding body of research indicates that VAT, as opposed to the overall body size, was a stronger predictor of cancer incidence [7] and all-cancer risks [8].

There are several ways that VAT is measured. Clinically, VAT is often assessed by MRI or CT. The ability to access medical imaging facilities limits its application to the general population [9]. While waist circumference (WC) and waist-hip ratio (WHR) are easily obtainable, they could not distinguish between VAT and trunk subcutaneous adipose tissue [10]. Therefore, integrated indices like visceral adiposity index (VAI) [11] and lipid accumulation product (LAP) [12] have been established to measure VAT. Both VAI and LAP have been applied in the context of diseases such as metabolic syndrome [13, 14] and type 2 diabetes mellitus (T2DM) [15–17]. However, these indices were developed based on specific ethnic populations, i.e., VAI from Italian [11] and LAP from Brazilian [12]. Limited information is available in terms of the ability of these indices to translate into the Asian population.

Chinese visceral adiposity index (CVAI) is a newly developed biomarker that considers the characteristics of body fat in Asians [18]. CVAI incorporated age, WC, body mass index (BMI), and blood lipid markers [18], and has been confirmed by CT for its relevance to VAT [18]. Studies have demonstrated associations between CVAI and the incidence of T2DM [19, 20], hypertension [20, 21], nonalcoholic fatty liver disease [22], and other chronic diseases[23, 24]. In some cases, CVAI exhibited superior predictive performance in disease prognosis compared to BMI, WC, and WHR [19, 25, 26]. Considering CVAI as a more suitable index of VAT in the Asian population, we wondered whether the index would be a better VAT biomarker for predicting cancer risk in Chinese populations. Therefore, we aimed to assess the associations of VAT biomarkers with cancer incidence in the MJ cohort, a large prospective cohort in Taiwan. We further compared CVAI with other VAT measurements, such as VAI, LAP, WC, and WHR.

## Methods

### Study population

**Fig. 1:**
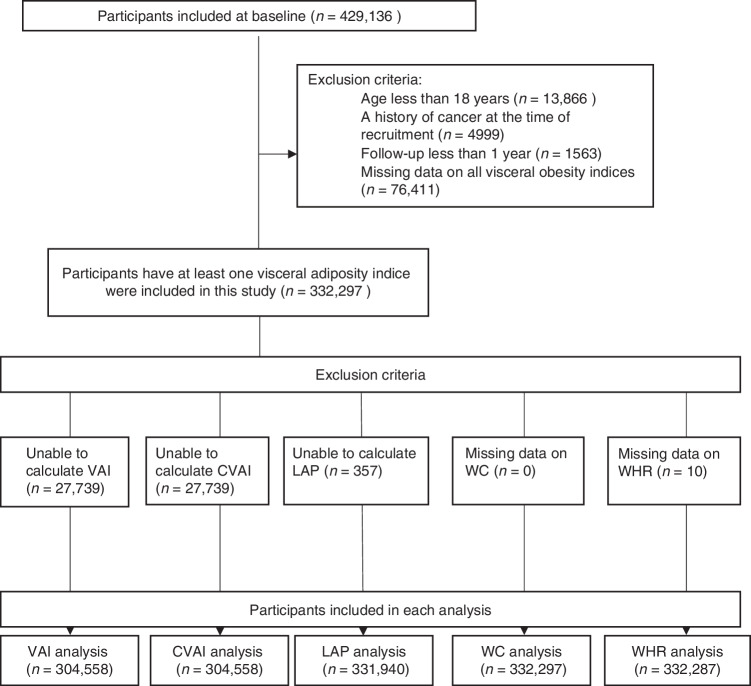
Participants included in this study. n sample size, VAI visceral adiposity index, CVAI Chinese visceral adiposity index, LAP lipid accumulation product, WC waist circumference, WHR waist-hip ratio.

Our study cohort was derived from the ongoing Taiwan MJ cohort study. This longitudinal study has enrolled participants who have undergone extensive medical screenings throughout Taiwan since 1996. Each individual within the MJ cohort completed questionnaires and underwent physical examinations. Additionally, they provided blood and urine samples for laboratory testing. The exclusion criteria for this study comprised individuals under the age of 18 (*n* = 13,866), those who had previously been diagnosed with cancer when enrolled (*n* = 4999), and those with a follow-up period of less than one year. (*n* = 1563). The study included 408,708 participants, with an average follow-up period of 6.7 years, varying from 1 to 11 years. Each adult was required to possess at least one of the five specific targeted visceral obesity indices (VAI, CVAI, LAP, WC, WHR). The ultimate study cohort comprised 332,297 participants, details regarding the selection procedure were illustrated in Fig. 1.

### Data collection

Trained staff collected data on sociodemographic characteristics (including age, sex, educational attainment, occupation, and marital status), lifestyles (such as smoking, drinking, and physical activity), as well as personal and family cancer history through questionnaires. Measurements for height, weight, WC, and hip circumference (HC) were documented. Moreover, laboratory tests were conducted on a fasting blood sample obtained during enrollment, encompassing the analysis of TG and HDL.

### Definition of visceral obesity indices

Three novel indices (i.e., VAI, CVAI, and LAP) have been developed by previous studies [11, 12, 18] to estimate VAT. These indices provided a more nuanced understanding of visceral obesity by incorporating various factors such as WC, BMI, TG, and HDL. The formulas for males and females are as follows:

For males:

$${{{\rm{VAI}}}}= 	{{{\rm{WC}}}}\,({{{\rm{cm}}}})/[39.68+1.88\times {{{\rm{BMI}}}}\,({{{\rm{kg}}}}/{{{{\rm{m}}}}}^{2})] \\ 	 \times [{{{\rm{TG}}}}\,({{{\rm{mmol}}}}/{{{\rm{L}}}})/1.03]\times [1.31/{{{\rm{HDL}}}}\,({{{\rm{mmol}}}}/{{{\rm{L}}}})]$$

$${{{\rm{CVAI}}}}=-267.93+0.68\times {{{\rm{age}}}}({{{\rm{y}}}})+0.03\times {{{\rm{BMI}}}}\,({{{\rm{kg}}}}/{{{{\rm{m}}}}}^{2})+4.00\times {{{\rm{WC}}}}({{{\rm{cm}}}})$$

$${{{\rm{LAP}}}}=[{{{\rm{WC}}}}\,({{{\rm{cm}}}})-65]\times {{{\rm{TG}}}}\,({{{\rm{mmol}}}}/{{{\rm{L}}}})$$

For females:

$${{{\rm{VAI}}}}= \, 	{{{\rm{WC}}}}\,({{{\rm{cm}}}})/[36.58+1.89\times {{{\rm{BMI}}}}\,({{{\rm{kg}}}}/{{{{\rm{m}}}}}^{2})] \\ 	 \times [{{{\rm{TG}}}}({{{\rm{mmol}}}}/{{{\rm{L}}}})/0.81]\times [1.52/{{{\rm{HDL}}}}\,({{{\rm{mmol}}}}/{{{\rm{L}}}})]$$

$${{{\rm{CVAI}}}}= 	-187.32+1.71\times {{{\rm{age}}}}({{{\rm{y}}}})+4.32\times {{{\rm{BMI}}}}\,({{{\rm{kg}}}}/{{{{\rm{m}}}}}^{2}) +1.12\times {{{\rm{WC}}}}\,({{{\rm{cm}}}}) \\ 	 +39.76\times {{{\rm{LgTG}}}}({{{\rm{mmol}}}}/{{{\rm{L}}}})-11.66\times {{{\rm{HDL}}}}\,({{{\rm{mmol}}}}/{{{\rm{L}}}})$$

$${{{\rm{LAP}}}}=[{{{\rm{WC}}}}\,({{{\rm{cm}}}})-58]\times {{{\rm{TG}}}}\,({{{\rm{mmol}}}}/{{{\rm{L}}}})$$

WHR is computed by dividing WC by hip circumference (HC). In this study, participants were initially categorized into two groups based on the WHO-recommended cut-off values for Asian populations for WC and WHR. Then, they were further categorized into four groups as categorical variables, determined by the median value of each subgroup [26–28]. In Asian populations, the WC cutoff is 90 cm for males and 80 cm for females. Similarly, the WHR cutoff is 0.9 for males and 0.85 for females. Individuals whose WC and WHR exceeded the defined cutoff points were identified as having central obesity. WC and WHR were further classified into four ascending groups according to the mentioned cutoff points, with Group 1 representing the lowest measurement.

### Study covariables

Occupation (blue collar, white collar, self-employed, and others), marital status (single, married, separated or divorced or widowed), educational attainment (middle school or below, high school, junior school, college or higher), drinking (never, former, or current), smoking (never, former, or current), and family cancer history (yes, no) were modeled as categorical variables. BMI was divided into four categories following a Chinese standard [29]: <18.49, 18.5–23.9, 24–27.9, and ≥28 kg/m^2^. Leisure-time physical activity was determined by multiplying intensity (metabolic equivalent, MET) and exercise duration (hours), and was grouped into four categories [30]: inactive (<3.75 MET hrs /week), low (3.75–7.49 MET hrs/week), medium (7.5–16.49 MET hrs/week), and high (≥16.5 MET hrs/week).

### Ascertainment of all-cancer incidence and cancer-specific incidence

The primary outcome evaluated was the overall cancer incidence. The follow-up period was determined from the date of recruitment to December 31, 2007, date of death, date of cancer incidence, or loss to follow-up, whichever came first. Information regarding follow-up procedures has been previously documented [30, 31].

We gathered data on cancer incidence by connecting participants in our study to both the Taiwan Cancer Registry and the Taiwan Death File from 1996 to 2007. This linkage was established through the unique IDs. Comprehensive details regarding the study design of the MJ cohort are available in other publications [31–33]. Cancer cases were based on the International Classification of Diseases, Ninth Revision (ICD-9) codes (140-239), and the International Classification of Diseases, Tenth Revision (ICD-10) codes (C00 to C97).

### Statistical analysis

Continuous variables were described as medians (interquartile ranges), and categorical variables as counts (percentages). Baseline characteristics were summarized by sex and compared between male and female participants using the χ^2^ test or Mann–Whitney U test. To address missing data, which accounts for 3% of the dataset, we generated 5 imputed data sets with 10 iterations and pooled the results using the R package “mice.”

VAI, CVAI, and LAP were used to divide participants into five groups based on ascending quintiles, labeled as Quintiles 1 through 5. We calculated the incidence rates per 100,000 person-years.

We compared the overall cancer incidence in participants with different visceral obesity indices using log-rank tests. Cox proportional hazard models were employed to compute hazard ratios (HRs) and their 95% CIs. The model was applied in males and females separately and was adjusted for age, educational attainment, marital status, occupation, drinking, smoking, physical activity, BMI, and family cancer history. We tested for trend by treating the categorical visceral obesity indices as continuous variables in the model (*P* for trend). The *P* values for interaction were computed through the comparison of the likelihood of fitted models, considering both with and without interaction terms.

Stratified analyses were applied to investigate the associations of smoking (never vs former or current) and drinking (never vs former or current) with potential associations between sex-specific visceral obesity indices and overall cancer incidence. All covariates mentioned above were adjusted except the study characteristic of the subgroup. Additionally, a cancer-specific analysis was performed for both males and females to explore the correlation between VAT indices and the incidence of specific cancers.

A two-sided *P*  <  0.05 was considered statistically significant. All analyses were performed using SAS 9.3 (SAS Institute, Cary, NC) and R software (version 4.0.4).

## Results

### Baseline characteristics of the population

The study encompassed 332,297 Chinese participants without a history of cancer, the average age was 40.3 years, and comprised 160,275 (48.2%) males and 172,022 (51.8%) females. During the follow-up period, 6879 cases of cancer were reported. Additional information on population characteristics is available in Table 1.

**Table 1:** Characteristics of study population.

	Male	Female	All	*P* value
	(*n* = 160,275)	(*n* = 172,022)	(*n* = 332,297)	
Age (yrs)				
Mean (SD)	40.3 (13.7)	40.2 (13.7)	40.3 (13.7)	0.026
Marital status				
Single	43,456 (27.1%)	43,157 (25.1%)	86,613 (26.1%)	<0.001
Married	104,018 (64.9%)	103,780 (60.3%)	207,798 (62.5%)	
Separated/divorced/widowed	4549 (2.8%)	16,098 (9.4%)	20,647 (6.2%)	
Missing	8252 (5.1%)	8987 (5.2%)	17,239 (5.2%)	
Education				
Middle school or lower	28,523 (17.8%)	49,268 (28.6%)	77,791 (23.4%)	<0.001
High school	34,674 (21.6%)	41,420 (24.1%)	76,094 (22.9%)	
Junior school	36,376 (22.7%)	34,818 (20.2%)	71,194 (21.4%)	
College or higher	54,307 (33.9%)	39,881 (23.2%)	94,188 (28.3%)	
Missing	6395 (4.0%)	6635 (3.9%)	13,030 (3.9%)	
Occupation				
White collar	14,437 (9.0%)	19,076 (11.1%)	33,513 (10.1%)	<0.001
Blue collar	45,390 (28.3%)	14,472 (8.4%)	59,862 (18.0%)	
Self employed	64,916 (40.5%)	105,810 (61.5%)	170,726 (51.4%)	
Other	26,579 (16.6%)	22,787 (13.2%)	49,366 (14.9%)	
Missing	8953 (5.6%)	9877 (5.7%)	18,830 (5.7%)	
Smoking status				
Never	75,564 (47.1%)	148,959 (86.6%)	224,523 (67.6%)	<0.001
Former or current	77,408 (48.3%)	13,401 (7.8%)	90,809 (27.3%)	
Missing	7303 (4.6%)	9662 (5.6%)	16,965 (5.1%)	
Drinking status				
Never	102,850 (64.2%)	145,567 (84.6%)	248,417 (74.8%)	<0.001
Former or current	48,155 (30.0%)	11,038 (6.4%)	59,193 (17.8%)	
Missing	9270 (5.8%)	15417 (9.0%)	24687 (7.4%)	
Physical activity (MET hours/week)				
<3.75	62,617 (39.1%)	91,261 (53.1%)	153,878 (46.3%)	<0.001
3.75–7.5	38,982 (24.3%)	36,852 (21.4%)	75,834 (22.8%)	
7.5–16.5	28,675 (17.9%)	22,898 (13.3%)	51,573 (15.5%)	
≥16.5	20,258 (12.6%)	10,815 (6.3%)	31,073 (9.4%)	
Missing	9743 (6.1%)	10,196 (5.9%)	19,939 (6.0%)	
BMI (kg/m^2^)				
18.5–24	80,577 (50.3%)	103,707 (60.3%)	184,284 (55.5%)	<0.001
<18.5	6961 (4.3%)	23,348 (13.6%)	30,309 (9.1%)	
24–28	56,256 (35.1%)	33,123 (19.3%)	89,379 (26.9%)	
≥28	16,449 (10.3%)	11,820 (6.9%)	28,269 (8.5%)	
Missing	32 (0.0%)	24 (0.0%)	56 (0.0%)	
Family history of cancer				
No	119,864 (74.8%)	123,732 (71.9%)	243,596 (73.3%)	<0.001
Yes	40,411 (25.2%)	48,290 (28.1%)	88,701 (26.7%)	
Visceral adiposity index				
Median [Q1, Q3]	1.29 [0.801,2.11]	1.04 [0.690,1.71]	1.15 [0.735,1.91]	<0.001
Missing	15,492 (9.7%)	12,247 (7.1%)	27,739 (8.3%)	
Chinese visceral adiposity index				
Median [Q1, Q3]	70.8 [39.0,101]	32.3 [1.42,75.2]	53.2 [15.7,90.6]	<0.001
Missing	15,492 (9.7%)	12,247 (7.1%)	27,739 (8.3%)	
Lipid accumulation product				
Median [Q1, Q3]	20.6 [9.94,38.4]	10.4 [5.06,22.2]	14.7 [6.58,30.6]	<0.001
Missing	167 (0.1%)	190 (0.1%)	357 (0.1%)	
Waist circumstance (cm)				
Median [Q1, Q3]	82.0 [76.0,88.0]	70.0 [65.0,77.0]	76.0 [69.0,84.0]	<0.001
Waist-hip ratio				
Median [Q1, Q3]	0.860 [0.818,0.902]	0.758 [0.721,0.802]	0.809 [0.750,0.870]	<0.001
Missing	3 (0.0%)	7 (0.0%)	10 (0.0%)	

*P* values for categorical variables were from Chi-square analysis, *P* value for continuous variables were from Student’s *t* test.

*n* sample size, *MET* metabolic equivalent, *BMI* body mass index, *SD* Standardized deviation, *Q1* the first quantile, *Q3* the third quantile.

### Associations between VAT indices and overall cancer incidence in males and females

**Fig. 2:**
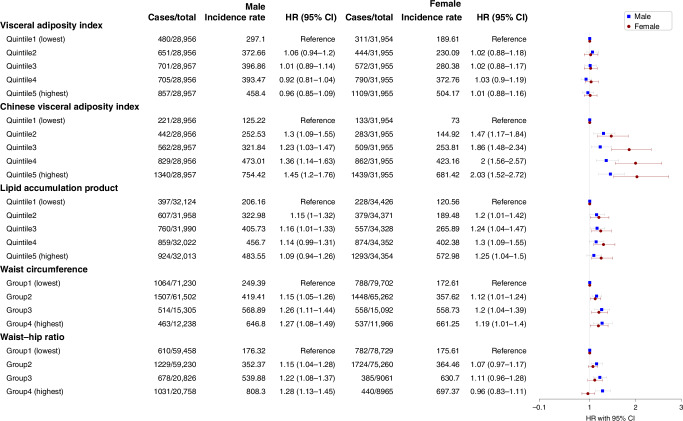
Sex-specific associations between visceral obesity indices and overall cancer incidence. The incidence rate was defined as 100,000 person-years. HR hazard ratio, CI confidence interval; HRs and CIs were from Cox regression models. Marital status, education, occupation, smoking status, drinking status, physical activity (MET-hours/week), BMI (kg/m^2^), and family history of cancer were adjusted in both the main analysis and trend analysis.

Based on the log-rank analysis, all five indices exhibited a positive correlation with the overall cancer incidence (all *P* < 0.001). Figure 2 shows the HRs for overall cancer incidence based on VAT indices after adjusting for confounders. The multivariable Cox regression models indicated that CVAI levels exhibited a dose-response relationship with overall cancer incidence in both men and women (Both *P* for trend <0.005). Compared to the Quintile 1 (lowest), the HRs for males were 1.3 (95% CI: 1.09–1.55), 1.23 (95% CI: 1.03–1.47), 1.36 (95% CI: 1.14–1.63), and 1.45 (95% CI: 1.2–1.76) for Quintiles 2, 3, 4, and 5, respectively. For females, the HRs were 1.47 (95% CI: 1.17–1.84), 1.86 (95% CI: 1.48–2.34), 2 (95% CI: 1.56–2.57), and 2.03 (95% CI: 1.52–2.72) for Quintiles 2–5 when compared to Quintile 1 (lowest).

In Fig. 2, no statistically significant associations were evident in either males or females when VAI was used as visceral obesity indices (all *P* > 0.05). For LAP, significant findings emerged among females when contrasted with Quintile 1 (lowest). Specifically, for Quintiles 2–5, HRs were 1.2 (95% CI: 1.01–1.42), 1.24 (95% CI: 1.04–1.47), 1.3 (95% CI: 1.09–1.55), and 1.25 (95% CI: 1.04–1.5), respectively, and did not reveal a substantial linear trend effect (*P* for trend = 0.068). Conversely, no significant outcomes were identified in males for Quintile 5 within the LAP category in comparison to Quintile 1 (lowest). Higher WC or WHR were associated with higher risks of cancer among males (both *P* for trend <0.005). The HRs for Group 4 in terms of WC and WHR were 1.27 (95% CI: 1.08–1.49) and 1.28 (95% CI: 1.13–1.45), respectively, when compared to Group 1. Among females, a significant correlation was observed with WC (HR:1.19, 95% CI: 1.01–1.4, *P* for trend = 0.031), but not with WHR (HR:0.96, 95% CI: 0.83–1.11, *P* for trend = 0.567) (Fig. 2).

### Interaction analysis of VAT indices with overall cancer incidence

**Fig. 3:**
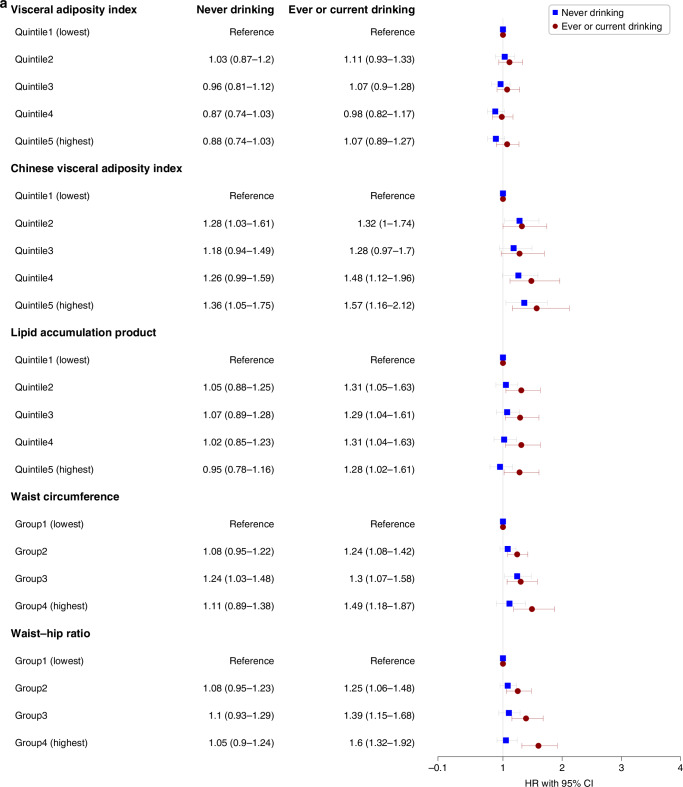

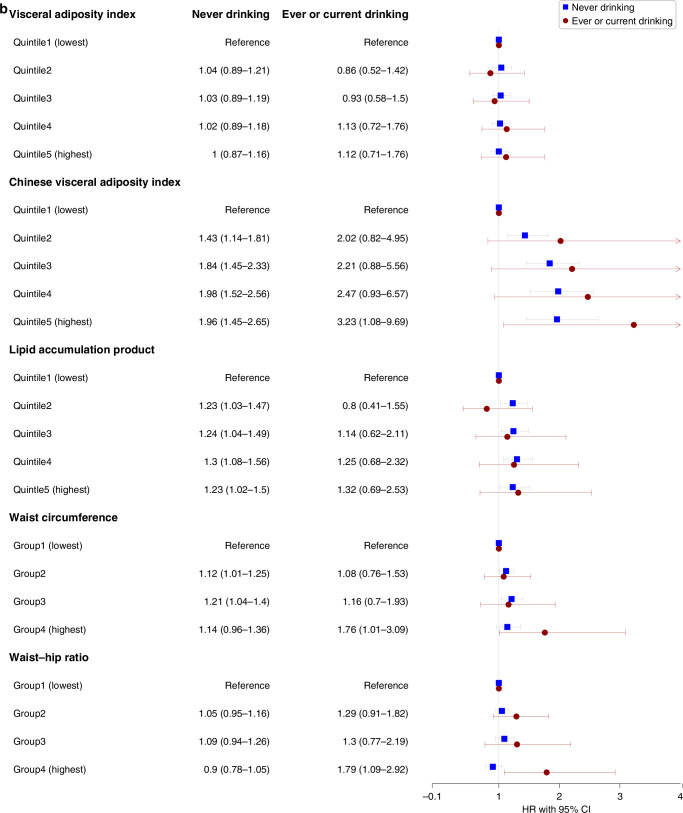
Sex-specific associations between visceral obesity indices and overall cancer incidence in different populations with different drinking status. **a** Associations in males. **b** Associations in females. HR hazard ratio, CI confidence interval. Stratified analyses were conducted to examine whether the potential association between sex-specific visceral obesity indices and overall cancer incidence differed by drinking status (never vs former or current). All covariates including marital status, education, occupation, smoking status, physical activity (MET-hours/week), BMI (kg/m^2^), and family history of cancer were adjusted.

**Fig. 4:**
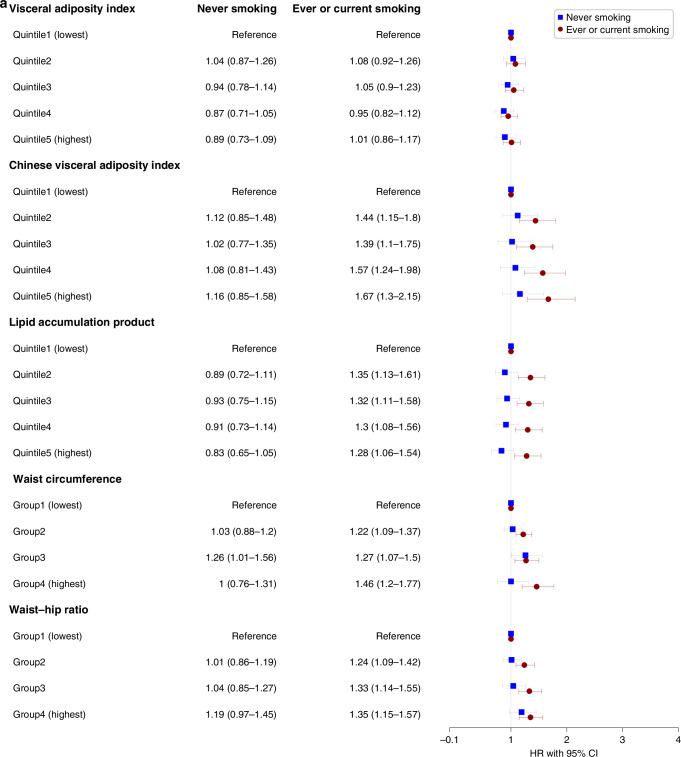

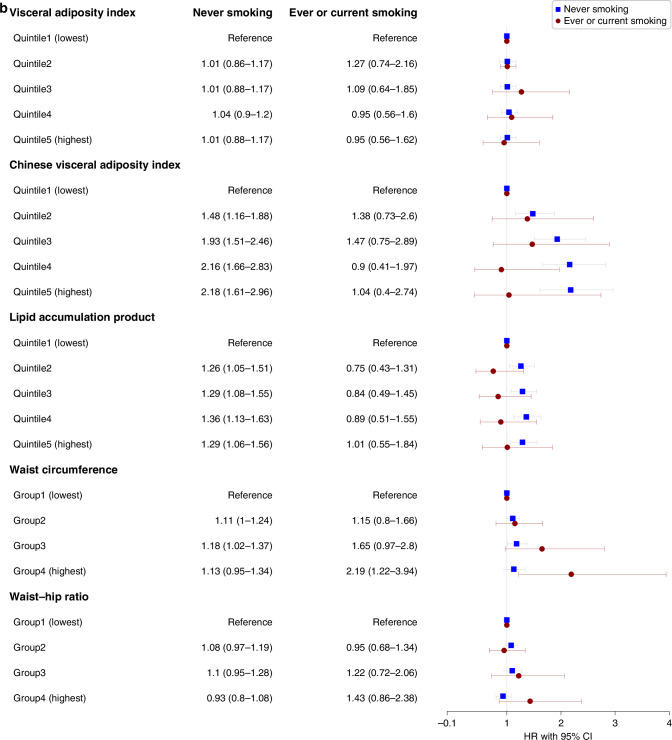
Sex-specific associations between visceral obesity indices and overall cancer incidence in different populations with different smoking status. **a** Associations in males. **b** Associations in females. HR hazard ratio, CI confidence interval. Stratified analyses were conducted to examine whether the potential association between sex-specific visceral obesity indices and overall cancer incidence differed by drinking status (never vs former or current). All covariates including marital status, education, occupation, drinking status, physical activity (MET-hours/week), BMI (kg/m^2^), and family history of cancer were adjusted.

We assessed the interaction between VAT indices and smoking status as well as drinking status in the adjusted model for the risks of overall cancer incidence in males and females, respectively (Figs. 3 and 4). We found interactions among CVAI, WC, WHR, and smoking status in males (all *P* < 0.05), indicating the associations in ever and current-smoking males were more evident. And no interactions were found in females.

### Analysis of VAT indices and cancer-specific incidence

**Fig. 5:**
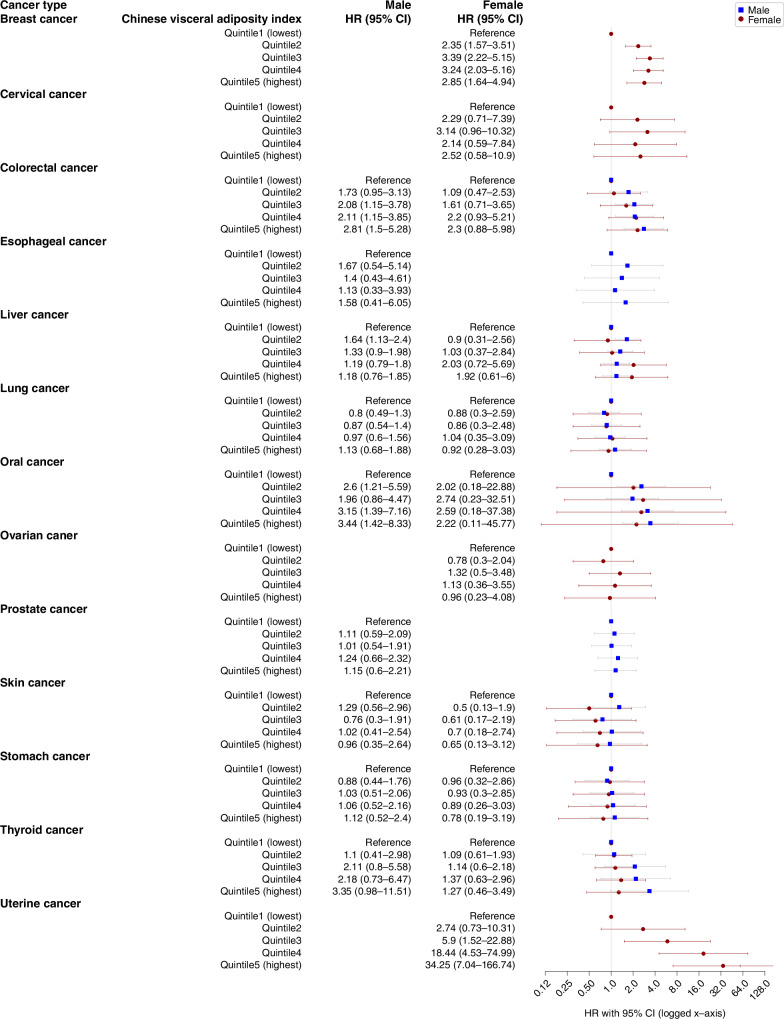
Sex-specific associations between Chinese visceral adiposity index and 13 types of cancer incidences. The incidence rate was defined as 100,000 person-years. HR hazard ratio, CI confidence interval, HRs and CI*s* were from Cox regression models. Marital status, education, occupation, smoking status, drinking status, physical activity (MET-hours/week), BMI (kg/m^2^), and family history of cancer were adjusted in both the main analysis and trend analysis.

Regarding cancer-specific incidences, our analysis revealed a noteworthy link between CVAI and heightened risks of colorectal and oral cancers in males. Specifically, when comparing Quintile 5 to Quintile 1 (lowest) of CVAI, the HR for colorectal cancer was 2.81 (95% CI: 1.5–5.82), and for oral cancer, the HR was 3.44 (95% CI: 1.42–8.33). Similarly, among females, CVAI demonstrated a connection with higher risks of breast and uterine cancers. Specifically, when comparing Quintile 5 to Quintile 1 (lowest) of CVAI, the HR for breast cancer was 2.85 (95% CI: 1.64–4.94), and for uterine cancer, the HR was 34.25 (95% CI: 7.04–166.74) (Fig. 5).

Supplementary Figs. 1 and 2 showed our analysis of cancer-specific incidences concerning WC and WHR. Like CVAI, we found notable associations primarily in colorectal, oral, lung, breast, and uterine cancer incidences. However, the results varied slightly between the different indices. WC was positively associated with lung cancer in males (Group 4 vs Group1, HR: 1.62, 95% CI: 1.17–3.03) and uterine cancer incidence in females (Group 4 vs Group1, HR: 2.63, 95% CI: 1.08–6.43), but was negatively associated with breast cancer in females (Group 4 vs Group1, HR: 0.66, 95% CI: 0.47–0.93; Supplementary Fig. 1). WHR was positively associated with colorectal cancer in both males (Group 4 vs Group1, HR: 1.65, 95% CI: 1.17–2.32) and females (Group 4 vs Group1, HR: 1.76, 95% CI: 1.15–2.7). WHR was also associated with lung (Group 4 vs Group1, HR: 1.55, 95% CI: 1.11–2.16) and oral cancer (Group 4 vs Group1, HR: 2.39, 95% CI: 1.39–4.09) in males. Different from CVAI but coherent with WC, a negative association was also observed between breast cancer and WHR (Group 4 vs Group1, HR: 0.57, 95% CI: 0.41–0.78) (Supplementary Fig. 2).

## Discussion

Our results validated the positive correlation between VAT and the overall cancer incidence within the large prospective MJ cohort. The findings further underscored the significance of CVAI’s association with overall cancer incidence in both sexes. In addition, CVAI was identified as an indicator for colorectal and oral cancer in males, breast and uterine cancer in females. CVAI demonstrates strong clinical utility as a predictor of cancer incidence in Chinese populations, outperforming both traditional anthropometrics (WC, WHR) and modern composite indices (VAI, LAP). CVAI could serve as a practical tool for cancer risk assessment in health check-up settings. Further validation in diverse populations would help confirm its generalizability.

Similar to our results, previous research has also indicated a link between elevated VAT levels and cancer incidence [7, 34–36]. A cohort comprising 68,253 Chinese women reported that they did not find any significant associations between WHR and overall cancer incidence after an adjustment of baseline BMI [37]. A cohort included 22.9 million Korean adults with 769,871 cancer cases found that WC quintiles were associated with cancer incidents in both males and females as well [38].

Despite WC and WHR, comprehensive indices like VAI and LAP have been used in cancer studies. However, many studied mortalities instead of cancer incidence. Within the UK Biobank, a prospective cohort including 357,457 individuals aged 38–73 years, the association between VAI and higher risks of all-cause and cause-specific mortalities was observed [39]. In 82,091 participants aged over 65 from the National Health and Nutrition Examination Survey, VAI levels were found to have a J-shaped relationship with all-cause mortality [40]. In the Tehran Lipid and Glucose Study, encompassing 6751 participants aged 30 years or older, mortality rates did not exhibit variance across LAP quantiles [41]. Although lacking evidence on cancer incidence, we suppose that VAI and LAP might not be accurate VAT predictors in the Chinese population, since they were formulated based on Western populations.

CVAI has been introduced as a proper VAT measurement in Chinese. In our study, CVAI showed better predictive value than VAI, likely due to the differing body fat characteristics among ethnicities. Previous studies have linked CVAI with various diseases, including coronary heart disease [23], cerebrovascular disease, diabetic kidney disease [42], and nonalcoholic fatty liver disease [43]. The link between CVAI and cancer incidence had been significantly under-researched, and our study addressed this gap.

Cigarette smoking has shown a positive association with central adiposity [44, 45]. In our study, the interactive analysis revealed stronger associations between overall cancer incidence and CVAI, WC, and WHR in males who were either current or former smokers, while no significant interaction was observed in females. Endogenous estrogen may attenuate the impact of visceral adiposity by modulating adipokine profiles and reducing inflammation. Visceral fat promotes carcinogenesis by secreting pro-inflammatory cytokines, which might interact with smoking-induced DNA damage, amplifying cancer risk [46, 47]. Interestingly, estrogen has been shown to mitigate adipose-related inflammation and metabolic dysfunction, potentially explaining the weaker VAT-cancer link in females [48]. Given our female cohort’s median age of 40.3 years, many likely retained estrogen’s protective effects. Despite sex differences, varying risk effects of VAT and smoking on cancer incidence have been reported. In a 7-year study of 22.9 million Korean adults, WC showed continued significance in cancers related to smoking, like oral and laryngeal cancers in males. However, its significance diminished in other cancers, such as brain cancer [49]. Furthermore, a study involving 162,679 males and females reported that WC was not linked to lung cancer within any smoking group after adjusting for BMI [50]. The diversity in cancer types across studies might contribute to the differing effects observed between smokers and non-smokers.

In line with our cancer-specific findings, an analysis based on 245,009 females, among whom there were 5402 cases of breast cancer in the UK Biobank also revealed a protective trend related to WC concerning breast cancer incidence among premenopausal women [51]. Differently, a study involving 116 breast cancer cases and 226 controls found VAI positively associated with breast cancer [42]. Significantly, our study primarily involved premenopausal participants, whose fat distribution were different from postmenopausal women [52]. Hence, CVAI, rather than anthropometric measurements such as WC and WHR, is potentially a more proper VAT metric in premenopausal women.

This study possesses a few strengths. Primarily, we are the first study to identify the associations of VAT indices, especially CVAI with cancer incidence in the Chinese population. Second, the prospective study enrolled a substantial sample size, potentially rendering the results more representative of the general population. Third, our prospective study design, with baseline measurements of adiposity traits, minimizes the impact of reverse causation. Fourth, this study collected sufficient lifestyle co-variates to largely attenuate its confounding effect. Moreover, mortality data were sourced from death registry records, ensuring minimal unavailability of individuals for follow-up.

However, there are also some limitations. First, we did not include a comparison with imaging data, which is the gold standard for measuring VAT. Secondly, our findings, based on Chinese individuals with higher socioeconomic status undergoing routine physical exams, may not apply to other ethnic or socioeconomic groups.

## Conclusion

In summary, increased VAT levels were related to elevated overall cancer incidence in the MJ cohort. CVAI was a superior clinical marker for predicting cancer incidence in the Chinese population, surpassing other VAT indices like WC, WHR, VAI, and LAP.

## Supplementary information

Supplementary Information

## Data Availability

The Taiwan MJ Cohort is available to the worldwide research community and offers collaboration. Applicants for data access should contact the MJ Health Research Foundation at [http://www.mjhrf.org/]. Further inquiries can be directed to the corresponding author.
