# Supplementary material for: Chinese visceral adiposity index outperforms other obesity indexes in association with increased overall cancer incidence: findings from prospective MJ cohort study
Source: Br J Cancer. 2025 May 10;133(2):227–38. doi: 10.1038/s41416-025-03041-1 (PMC12304270; doi:10.1038/s41416-025-03041-1)
Supplement: Supplementary file 1 — Supplementary Information [file 41416_2025_3041_MOESM1_ESM.docx]

**
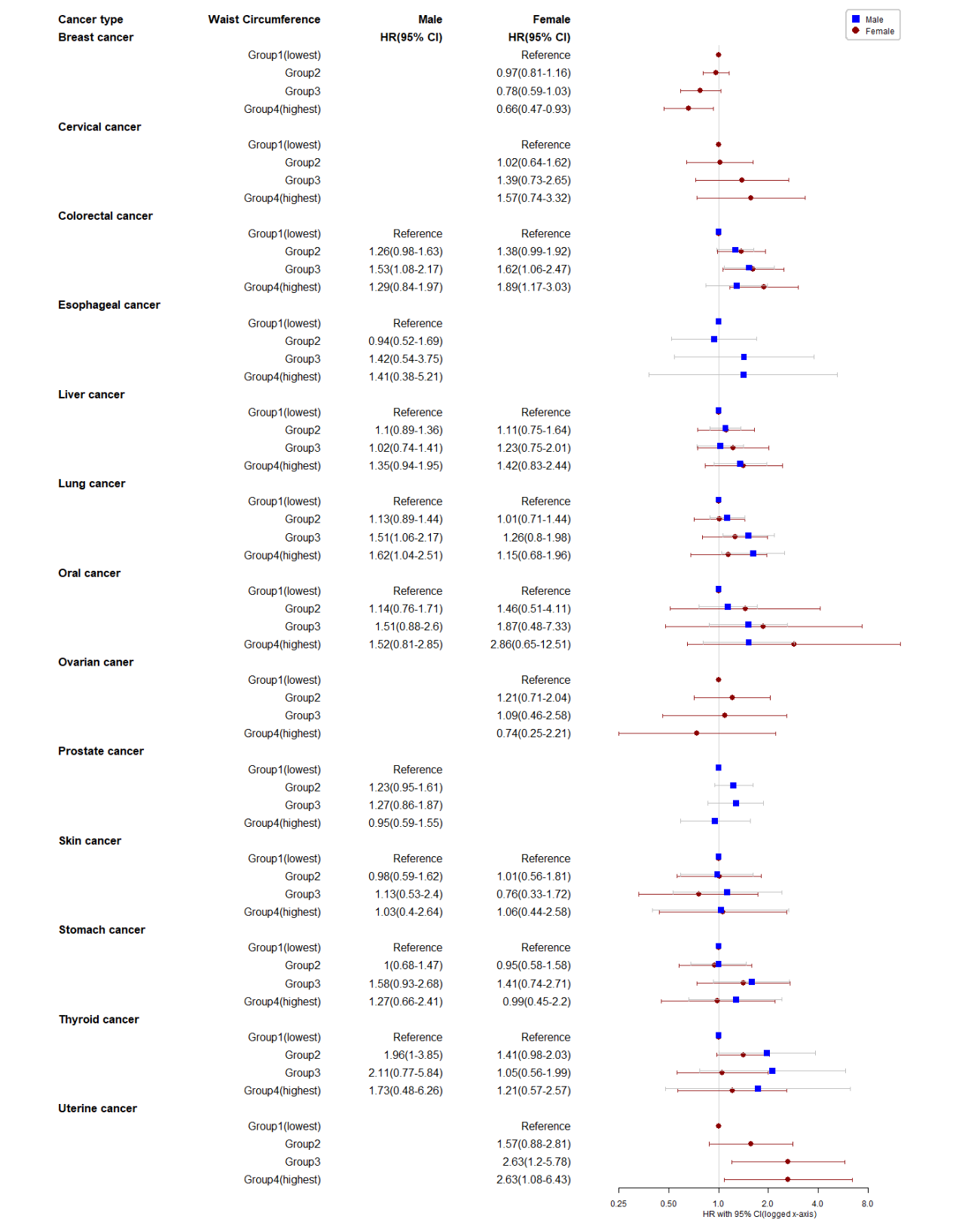
**

**Supplementary Fig. 1. Sex-specific associations between waist circumference and 13 types of cancer incidences.** Incidence rate was defined as 100,000 person years. HR: hazard ratio; CI: confidence interval; HRs and CI*s* were from Cox regression models. Marital status, education, occupation, smoking status, drinking status, physical activity (MET hours/week), BMI (kg/m2) and family history of cancer were adjusted in both main analysis and trend analysis.
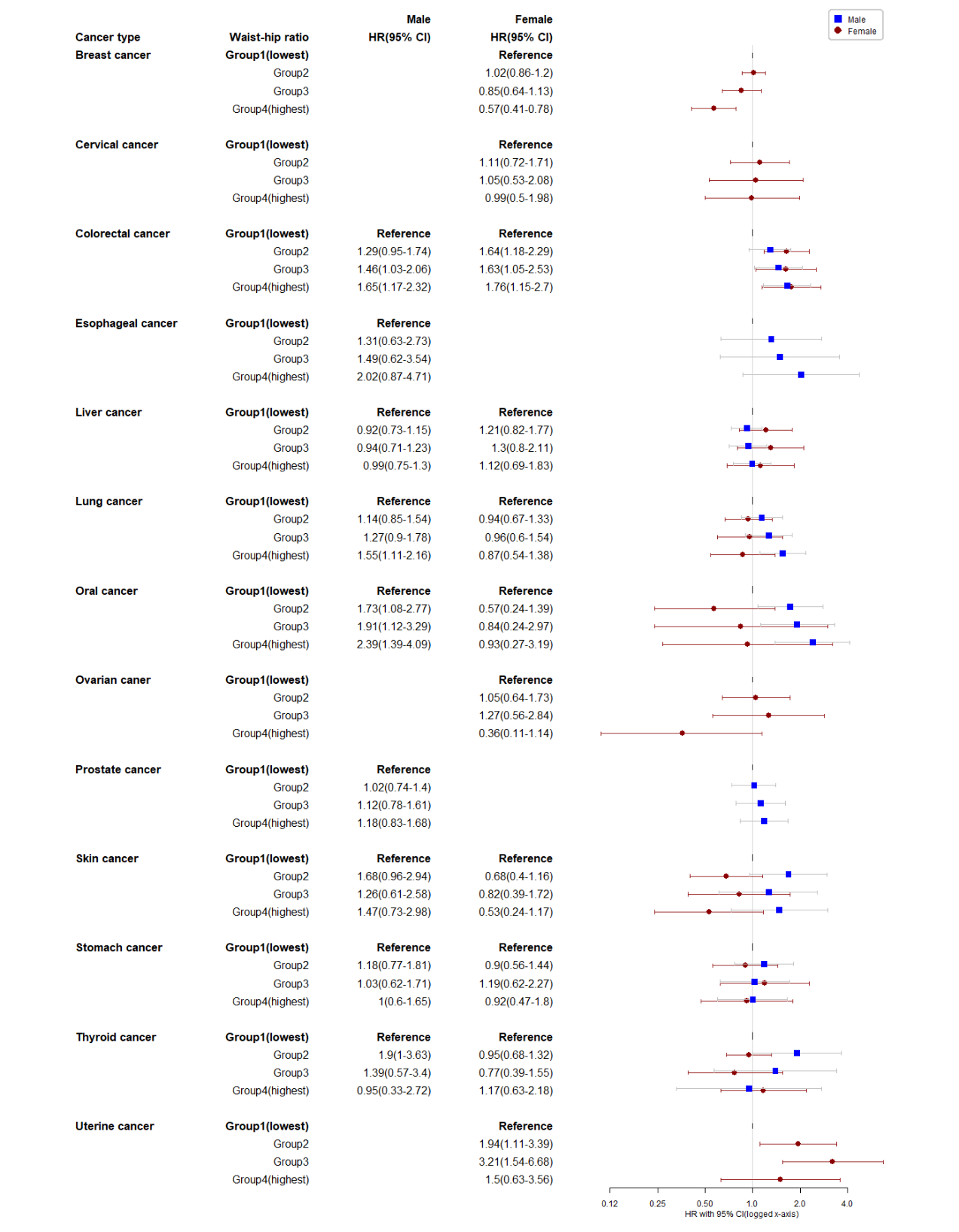


**Supplementary Fig 2. Sex-specific associations between waist-hip ratio and 13 types of cancer incidences.** Incidence rate was defined as 100,000 person years. HR: hazard ratio; CI: confidence interval; HRs and CI*s* were from Cox regression models. Marital status, education, occupation, smoking status, drinking status, physical activity (MET hours/week), BMI (kg/m2) and family history of cancer were adjusted in both main analysis and trend analysis.
